# Impaired Cardiorespiratory Fitness of Elite Athletes after Asymptomatic or Mild SARS-CoV-2 Infection

**DOI:** 10.3390/medicina60050786

**Published:** 2024-05-09

**Authors:** Tamara Stojmenović, Srdjan Marković

**Affiliations:** Faculty of Physical Education and Sports Management, Singidunum University, 11000 Belgrade, Serbia

**Keywords:** COVID-19, disease, sports, maximal oxygen consumption, ventilatory efficiency

## Abstract

*Background and Objectives:* The aim of the study was to evaluate the health status of professional athletes after recovering from COVID-19 and the impact that SARS-CoV-2 had on their overall cardiorespiratory fitness, which was done by conducting cardiopulmonary exercise testing (CPET). *Materials and Methods:* A total of twenty-seven professional basketball players (Euroleague Basketball and the ABA League) participated in the study. CPET was performed before (as part of their regular preparticipation exam, during the pre-season period), as well as after SARS-CoV-2 infection (after two weeks of home isolation, during the competitive part of the season). CPET was performed on a treadmill, while cardiovascular, respiratory, and metabolic functions were evaluated by using a breath-by-breath analysis technique (Quark CPET system manufactured by Cosmed, Rome, Italy). *Results:* Maximal oxygen consumption and aerobic efficiency were significantly reduced after SARS-CoV-2 infection (*p* = 0.000). An obvious decrease in oxygen pulse was observed during CPET after recovering from COVID-19 (*p* = 0.001), as was deterioration of ventilatory efficiency. Internal respiration was the most negatively affected. An early transition from aerobic to anaerobic mechanisms of creating energy for work and intensive metabolic fatigue were obvious after SARS-CoV-2 infection. *Conclusions:* Although it was believed that SARS-CoV-2 only affects the cardiopulmonary status of the elderly population and people with associated comorbidities, it is clear from this research that professional athletes can also be at certain risk. Even though no pathological cardiovascular and respiratory changes were found in athletes after COVID-19, results showed significantly decreased cardiorespiratory fitness, with an emphasis on internal respiration.

## 1. Introduction

Coronavirus disease 2019 (COVID-19) is an infectious disease caused by severe acute respiratory syndrome coronavirus 2 (SARS-CoV-2), which has taken the form of a pandemic since 2020. At the beginning of the pandemic, COVID-19 was considered a primary respiratory infection [1]. However, the course of the pandemic demonstrated that this type of virus can affect any organ system. In many cases, the cardiovascular, digestive, and nervous organ systems were compromised by COVID-19 [2,3,4]. Most infected people experienced mild to moderate illness and recovered without major complications. However, some became seriously ill and required serious medical attention, accompanied by severe and life-threatening complications. At first, it was reported that older people and those with underlying medical conditions were more likely to develop serious illness but, in the end, it turned out that anyone could get sick with COVID-19 and become seriously ill, or even die, at any age [5]. Furthermore, overweight individuals affected by COVID-19 were more likely to develop more severe acute disease and have a slower recovery of cardiopulmonary fitness and long-term symptoms. It was also shown that severe/critical cases maintained worse hemodynamic responses to exercise compared to mild ones considering general population [6].

Even though COVID-19 cases and deaths were lower when levels of physical activity were high [7], the ongoing pandemic did not spare even professional athletes in the sense of being infected with a virus and the possibility of developing cardiovascular problems and other post-COVID-19 sequalae [8]. One study showed that from 226 recruited elite university athletes, 53.5% of them returned to ordinary training immediately after quarantine, while 61.5% experienced a disturbance in ordinary training and 30.9% experienced that in competition after SARS-CoV-2 infection [9]. On the contrary, different research has reported that elite athletes maintain peak performance after testing positive for SARS-CoV-2 and that reported prevalence of clinical and subclinical myocarditis among competitive athletes recovering from COVID-19 is not very high [10]. Considering that athletes belong to a young and healthy population, it was initially believed that their health was not too threatened by COVID-19 infection. At the very beginning of the epidemic, return-to-play recommendations for athletes with or without mild symptoms included only routine physical examination and resting ECG, without more detailed diagnostic procedures or biochemical analyses [11,12]. However, it has been shown that even healthy, active individuals can also suffer from severe consequences of infection, even if they had suffered a mild version of the disease. Post-COVID-19 symptoms such as fatigue, coughing, chest pain, palpitations, dyspnea, and others were reported by many physically active people upon their return to training activities [8]. Also, athletes, together with the general population, could develop prolonged COVID-19 (long-haul) syndrome, which was most often characterized by prolonged fatigue and cardiopulmonary limitations during exertion [13]. Furthermore, cardiovascular problems caused by COVID-19 in the general population alerted medical experts to turn their attention toward the effect of the infection on the cardiovascular function of professional athletes. The main concern was the possibility of developing myocarditis as one of the causes of sudden cardiac death in sports [14]. The above-mentioned concerns indicated the need for more detailed medical examinations, which included transthoracic 2D echocardiography, cardiac magnetic resonance imaging, lung function examination, cardiopulmonary exercise testing, and a detailed biochemistry analysis. These medical examinations revealed a certain number of cases of myocarditis, pericarditis, severe heart rhythm disorders, and impaired lung function, due to which the athletes were forbidden from returning to sports for a given period [15,16]. Even though recent data suggest that there is a very low risk of clinically significant cardiac involvement in young, healthy athletes following asymptomatic or mild COVID-19 [8], the issue of a safe return to sports is still present. A multidisciplinary approach should be employed by medical staff, coaches, and the athletes themselves in terms of when and how to return to play without compromising the athlete’s health or provoking injury. In other words, both the health status and the functional capacity of athletes must be evaluated to safely return them to regular training sessions after SARS-CoV-2 infection [12,13]. Accordingly, the post-COVID-19 pre-participation protocol has changed in favor of more detailed medical examinations [14,17].

Considering all the above, the aim of the study was to evaluate the cardiorespiratory status of professional athletes after suffering from COVID-19, as well as the impact of SARS-CoV-2 on their cardiorespiratory fitness, by conducting cardiopulmonary exercise testing.

## 2. Materials and Methods

### 2.1. Participants and Study Design

A total of twenty-seven male professional basketball players (Euroleague Basketball and the ABA League) participated in the study. Athletes who had been infected with SARS-CoV-2 were recruited in the study to evaluate their cardiopulmonary function end level of cardiorespiratory fitness by performing a standard pre-participation medical exam, which included physical examination and electrocardiogram at rest, and cardiopulmonary exercise testing. Additionally, their cardiorespiratory fitness was compared to the level that had existed prior to contracting COVID-19. According to the sports law of Serbia, every athlete must undergo a standard pre-participation medical examination twice a year. Professional athletes are obliged to perform a maximum-effort test as part of a regular medical checkup. Given the new circumstances during the COVID-19 pandemic, all professional athletes who had been infected with SARS-CoV-2 were required to undergo a CPET before returning to regular sports activities.

An observational analytical study was conducted to establish an association between exposure to SARS-CoV-2 and cardiorespiratory fitness. Cardiopulmonary exercise testing (CPET) was performed before and after SARS-CoV-2 infection. CPET was conducted as a standard pre-participation medical examination in August 2020 (pre-season period) after a lockdown period (started on 15 March and ended on 6 May 2020). At this point, none of the athletes had reported SARS-CoV-2 infection (all of them had negative PCR tests prior to their medical check-up), and all examined athletes were cleared for sports participation. Post-COVID-19 CPET was performed on the same study participants, two weeks after their first disease symptoms and a positive PCR test, in the period between November 2020 and February 2021 (depending on when each individual athlete had tested positive for SARS-CoV-2). At the time of conducting the study, a minimum of 2 weeks of home isolation was advised by the government of Serbia for all individuals infected with SARS-CoV-2 to prevent the spread of the infection, regardless of whether it was an asymptomatic or mild form of the disease. These tests were conducted during the competition part of the season. At this point, none of the players have been vaccinated. All participants had asymptomatic or mild COVID-19 and went through fourteen days of home isolation without any medical treatment for their symptoms. Before the CPET, athletes had to pass the physical examination at rest (electrocardiogram, blood pressure measuring, lung and heart auscultation). Prior to post-COVID-19 testing, all participants also had to show normal values of cardiac biomarkers, such as CRP, D-dimer, NT-proBNP, and high-sensitivity cardiac troponin (hs-cTn), as well as transthoracic 2D echocardiography. These diagnostic procedures were carried out to make sure that there were no ongoing acute inflammatory processes or contraindications for performing CPET.

Written consent was obtained through a letter given to athletes explaining the risk of CPET examination and the study’s goals, procedures, and methods. The protocol was in accordance with the Declaration of Helsinki for research on human subjects and was approved by the Ethical Committee via protocol number 348. There was no funding for this research.

### 2.2. Instruments and Measurements

CPET, as a maximal symptom limited exercise test, was performed on a treadmill. According to the incremental protocol adopted for professional athletes [18], subjects were equipped with a face mask, a heart-rate monitor (COSMED Wireless HR Monitor, Rome, Italy), and an ECG device (Quarck T 12x, Wireless 12-lead ECG, Rome, Italy) to perform the test. The initial speed and inclination were set at 5 km/h and 3°, respectively. Every 60 s, the treadmill speed was increased by 1 km/h, and the inclination remained constant throughout the test. The test was considered maximal when participants reached three out of the four criteria for achieving maximal effort (subjective feelings of exhaustion, changes in oxygen consumption of less than 150 mL/kg/min near the end of the test (which meant that a plateau had been reached in oxygen consumption and that no further increase in VO_2_max was expected despite the increase in work intensity), more than 90% of maximum heart rate, and a respiratory exchange ratio greater than 1.10) [18]. Direct gas exchange was measured by using the breath-by-breath analysis technique (Quark CPET system manufactured by Cosmed, Rome, Italy). All the tests were performed by a sports medicine specialist, while volume and gas calibrations were carried out before each testing day.

#### 2.2.1. Electrocardiographic Monitoring

Continuous ECG monitoring was performed to evaluate heart conditions during physical activity, as well to obtain maximal heart rate at the end of the test and to assess the three-minute heart-rate recovery. Oxygen pulse (VO_2_/HR), as an indirect indicator of left ventricular function (or ejection fraction), was measured and assessed by the Quark CPET system and the Wasserman 9-Panel Plot (Cosmed, Rome, Italy).

#### 2.2.2. Respiratory Function

Respiratory frequency and breathing reserve were estimated at the ventilatory anaerobic threshold (VAT) and respiratory compensation point (RCP) to evaluate lung function during effort in terms of adequate oxygen delivery, economical oxygen consumption, and carbon dioxide elimination. Ventilatory efficiency was calculated using the VE/VCO_2_ index and oxygen uptake efficiency slope (OUES), which represents the relation between oxygen uptake and minute ventilation, using a breath-by-breath analysis technique.

#### 2.2.3. Aerobic Capacity and Metabolic Response to Effort

Maximal oxygen consumption (VO_2_max) as an objective and accurate indicator of cardiorespiratory fitness and aerobic endurance was evaluated at the end of CPET. Aerobic economy (efficiency) was calculated as the amount of oxygen (in milliliters) consumed by each athlete at a steady state of intensity and VAT. The mean value of oxygen consumption at speeds of 8 and 9 km/h was taken as an indicator of aerobic economy. At these speeds, the respiratory quotient (VCO_2_/VO_2_) was below 0.80, which means that the athletes used exclusively aerobic mechanisms to obtain energy for a steady state of exercise intensity. Heart rate was measured at VAT and RCP to assess the intensity at which everyone reached the aerobic and anaerobic thresholds. VAT and RCP were determined by Wasserman 9-Panel Plot. Additionally, the respiratory exchange ratio (RER), as a measure of exhaled carbon dioxide and inhaled oxygen (CO_2_/O_2_), was calculated at the end of the test to assess the metabolic response to maximal effort and level of anaerobic exertion. Heart rate at RER = 1 was measured and demonstrated the intensity of the exertion at which the onset of absolute anaerobic metabolism had begun [16,18].

### 2.3. Statistical Analysis

Statistical analysis was carried out using IBM SPSS Statistics software version 20.0. All data were assessed for normality by performing a one-sample Kolmogorov–Smirnov test. Paired samples of *t*-tests were used to compare the measured characteristics of basketball players before and after SARS-CoV-2 infection. Pearson’s correlation analysis was performed to investigate correlations between the variables. The following classification pattern for r-values was used to assess the significance of the correlations: ≤0.29, low or weak; 0.30 to ≤0.39, moderate; ≥0.40 to ≤0.69, strong or high; ≥0.7, very high; and 1, perfect [19]. A simple linear regression was performed to determine the ability of VO_2_/HR (mL/beat), OUES, ventilatory efficiency, and heart rates at VAT and RCP to predict VO_2_max value (mL/kg/min). Descriptive statistics of data are given as $\bar{X}$ ± SD. A *p*-value ≤ 0.05 was considered statistically significant. To evaluate the power of the study sample, G*Power 3.1 software was used. A post hoc calculation was performed for the results of the comparison between the pre- and post-COVID-19 CPET (n = 27). A two-way dependent *t*-test was used, with α = 0.05. Both the mean and standard deviations of the research groups (pre- and post-COVID-19 tests) for the variable VO_2_pred (%) for professional basketball players were used to evaluate the effect size [20] (pre-COVID-19 CPET: 93.1 ± 11.77% and post-COVID-19 CPET: 83.9 ± 9.87%). The calculated sample power was (1 − β) = 0.979.

## 3. Results

In total, 27 subjects participated in the study (age at the point of pre-COVID-19 test 24.2 ± 5.5 years; age at point of post-COVID-19 test 24.5 ± 5.7 years). Descriptive statistics considering measured variables before and after SARS-CoV-2 infection are presented in Table 1.

### 3.1. Cardiovascular Function

**Figure 1:**
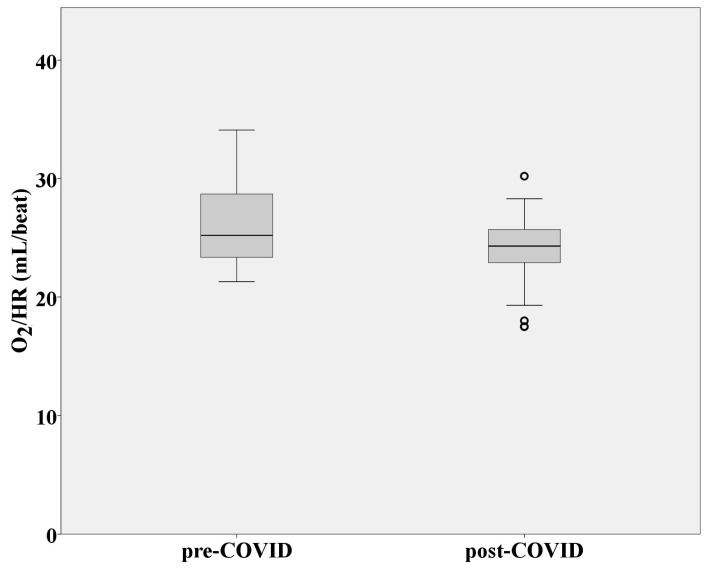
Distribution of oxygen pulse values in athletes before and after SARS-CoV-2 infection. Abbreviation: VO_2_/HR (mL/beat) = oxygen pulse.

CPET before and after SARS-CoV-2 infection did not show any pathological ECG changes considering rhythm and repolarization disorders. No statistically significant difference was observed in terms of achieved maximum heart rate and three-minute heart-rate recovery before and after SARS-CoV-2 infection (*p* > 0.05). Pre- and post-COVID-19 oxygen pulse values were statistically significant (*p* = 0.001). An obvious decrease in VO_2_/HR was observed during post-COVID-19 CPET (Figure 1).

### 3.2. Respiratory Function

**Figure 2:**
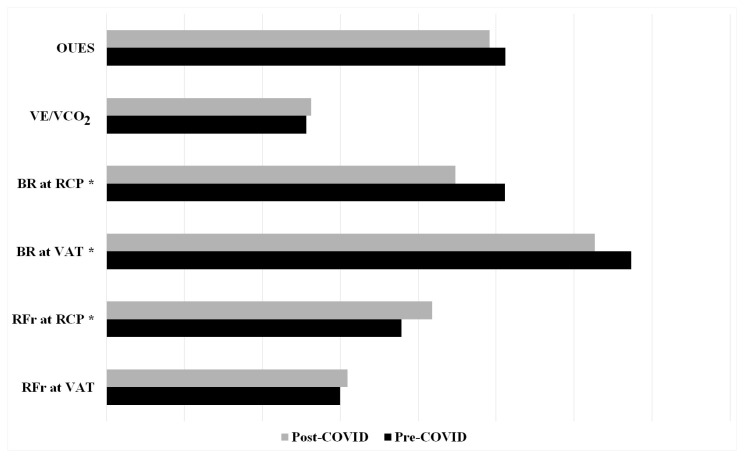
The influence of SARS-CoV-2 infection on specific markers of respiratory function of professional athletes. * *p* < 0.05. Abbreviations: BR = breathing reserve; OUES = oxygen uptake efficiency slope; RCP = respiratory compensation point; RFr = respiratory frequency; VAT = ventilatory anaerobic threshold; VE/VCO_2_ = ventilatory efficiency.

There were no statistically significant changes in respiratory frequency at VAT when comparing values obtained from pre- and post-COVID-19 CPET (*p* > 0.05). However, an increase in respiratory rates at RCP was observed (*p* = 0.041), as was a decrease in breathing reserves at both VAT and RCP during post-COVID-19 testing (*p* = 0.022 and *p* = 0.000, respectively). No statistically significant difference was observed in terms of ventilatory efficiency and OUES, even though there was an obvious numerical difference in terms of deteriorating ventilatory efficiency and decreasing OUES values (Figure 2).

### 3.3. Aerobic Capacity and Metabolic Response to Effort

**Figure 3:**
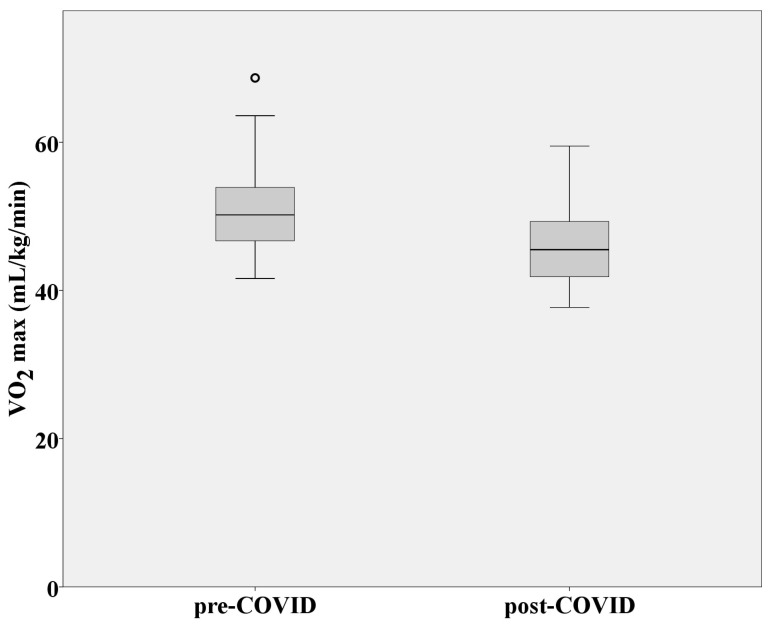
Distribution of VO_2_max values in athletes before and after SARS-CoV-2 infection. Abbreviation: VO_2_max = maximal oxygen consumption.

**Figure 4:**
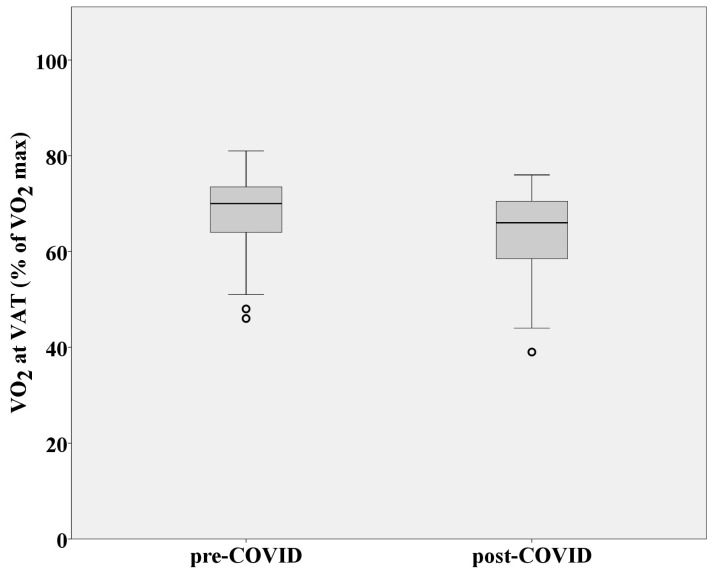
Oxygen consumption at VAT in athletes before and after SARS-CoV-2 infection. Abbreviation: VAT = ventilatory anaerobic threshold.

Maximal oxygen consumption as a measure of cardiorespiratory fitness and aerobic endurance was significantly reduced after SARS-CoV-2 infection (*p* = 0.000) (Figure 3). VO_2_ at VAT was also significantly decreased while performing CPET after COVID-19 (*p* = 0.000), which means that factors that affect oxygen delivery to tissues were influenced by infection (Figure 4). Aerobic efficiency was reduced because of COVID-19 in terms of uneconomic consumption of oxygen (VO_2_) during steady-state intensity. Mean VO_2_ during post-COVID-19 CPET at speeds of 8 and 9 km/h (steady-state intensity; respiratory quotient: CO_2_/O_2_ < 0.80; absolute aerobics) was statistically significantly higher compared to pre-COVID-19 CPET (*p* = 0.001). Greater amounts of oxygen had to be consumed at a given steady state of intensity due to earlier SARS-CoV-2 infection. Heart rates at VAT, RCP, and RER = 1 were considerably lower than values achieved during the pre-COVID-19 CPET (*p* = 0.021; *p* = 0.005, and *p* = 0.001, respectively). Maximal RER values (at the end of the test) were statistically significantly higher after performing post-COVID-19 CPET (*p* = 0.002). All this indicates an early transition from aerobic to anaerobic mechanisms of creating energy for work, as well as intense metabolic fatigue, to which the athletes were exposed because of a SARS-CoV-2 infection.

**Figure 5:**
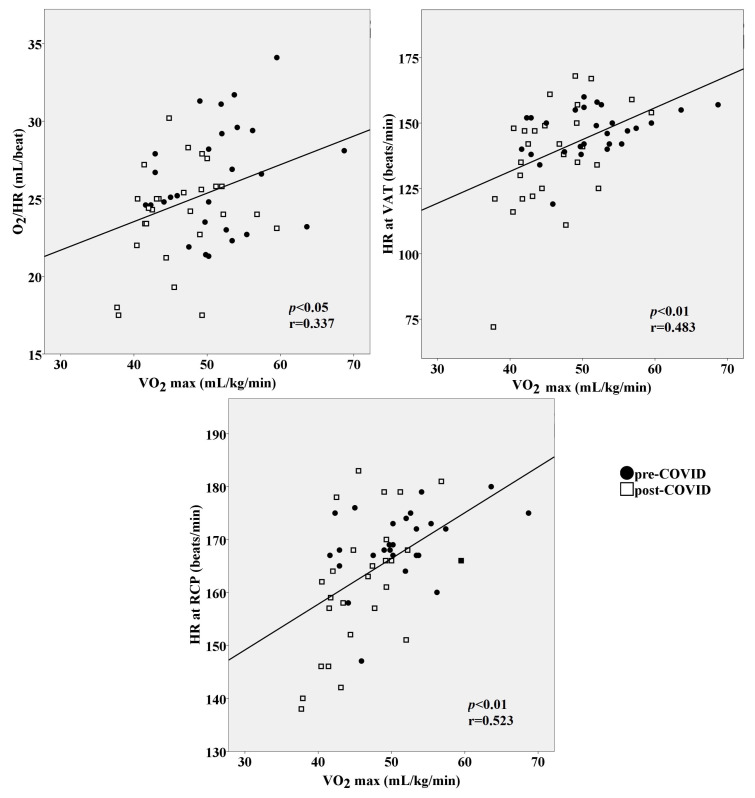
VO_2_max response profiles and their relationship with evaluated variables during cardiopulmonary exercise test. Pearson’s correlation analysis was performed to investigate correlations between variables. Abbreviations: HR = heart rate; VO_2_/HR = oxygen pulse; RCP = respiratory compensation point; VAT = ventilatory anaerobic threshold; VO_2_max = maximal oxygen uptake.

We also observed that reductions in VO_2_/HR, HR at VAT, and HR at RCP were all related to lower VO_2_max values at the end of CPET (r = 0.337 moderate, r = 0.483 strong, r = 0.523 strong magnitude of the correlations, *p* < 0.05, respectively) (Figure 5), demonstrating that the worse the O_2_ pulse and the lower the heart rates at VAT and RCP, the lower the maximal oxygen consumption. In addition, oxygen pulse, ventilatory efficiency, and an early transition from aerobic to anaerobic metabolic pathways altogether accounted for 34% of the variability in the VO_2_max response in athletes (Table 2).

## 4. Discussion

The objective of the study was to evaluate the health status of professional athletes after recovering from COVID-19 and the impact that SARS-CoV-2 had on their overall cardiorespiratory fitness, which was carried out via CPET. The results of the study indicated an obvious decline in the cardiorespiratory fitness of professional athletes after suffering from SARS-CoV-2 infection. Although none of the athletes had severe, clinical signs of disease, and there were no pathological cardiorespiratory changes during post-COVID-19 CPET, it was clear that the links in the chain responsible for the delivery and consumption of oxygen (upper and lower respiratory tract, myocardium, circulatory system, and muscle cells) with the aim of creating adenosine triphosphate (ATP) were under the negative influence of COVID-19, especially at the level of internal respiration.

Oxygen pulse, as an indirect indicator of left ventricular function [21], was significantly reduced after SARS-CoV-2 infection. Even though the recorded data, both before and after SARS-CoV-2 infection, were within the expected values for professional sports (>20 mL/beat) [22], the amount of oxygen delivered to the muscle cells with each heart contraction was significantly reduced after COVID-19. This result could potentially explain the overall decrease in cardiorespiratory fitness of basketball players, since the heart muscle is one of the most important links for delivering oxygen to the working muscles. Previous research reported that COVID-19 did not impact the sports performance of elite athletes [23,24]. However, these research data referred to individual examples of elite athletes and their peak results achieved several months after being infected with SARS-CoV-2. The results of our study were obtained after 14 days of home isolation, which may explain the reduced maximum oxygen consumption in the early stages of recovery after SARS-CoV-2 infection.

External respiration was not compromised by infection, which means that the diffusion of gases at the level of the respiratory membrane was adequate to satisfy the body’s needs for oxygen. It has been observed that one of the first objective indicators of the clinical signs of respiratory failure after COVID-19 include hypoxemia-associated changes in external respiration [25]. Since there were no pathological changes at the level of this part of the breathing process, it is obvious that respiratory function was not significantly damaged by COVID-19. This fact was confirmed by VE/VCO_2_ slope and OUES values, which did not change statistically significantly before and after SARS-CoV-2 infection. However, with increasing intensity during CPET, obvious increases in respiratory frequency and decreases in breathing reserves were observed at certain level of intensity after suffering from COVID-19. Apparently, a greater ventilation rate was needed to enable an adequate supply of oxygen and removal of carbon dioxide at the lung level. This fact could explain the slight increase in VE/VCO_2_ slope values, which represents the measure of ventilatory efficiency. Even though the pre- and post-COVID-19 data were within the normal values (VE/VCO_2_ between 20 and 30) [26], it was noticed that athletes had increased ventilatory requirements for a given level of exercise after SARS-CoV-2 infection. These findings agree with recent research on exertional intolerance after COVID-19, but among the non-athlete population (sedentary and recreational population) [27,28]. A follow-up to assess the long-term effects of SARS-CoV-2 infection on specific markers of respiratory function would be interesting for future research. The results of our study confirmed that even professional athletes were not spared from COVID-19 infection in terms of impaired cardiorespiratory function, which includes ventilatory efficiency. Furthermore, OUES, as an index of cardiorespiratory functional reserve [29], was numerically decreased after SARS-CoV-2 infection, which could explain the overall drop in exercise tolerance among athletes. Decreased values of OUES after COVID-19 coincided with an early transition from aerobic to anaerobic metabolism at submaximal levels of intensity. The exertional intolerance frequently observed following COVID-19 was most often described in patients who had survived a severe form of the disease, or among the recreational population [27,30,31]. Although the participants of our study had milder forms of disease and were professional athletes, the obtained results are in accordance with previous research on this topic [27,30,31].

Considering all the above (decreased VO_2_/HR, breathing reserve, and OUES; increased VE/VCO_2_ and respiratory frequency) a drop in VO_2_max was logical, but the question arose as to why there was a very pronounced decrease in aerobic capacity after only two weeks of rest and after asymptomatic or mild SARS-CoV-2 infection. Based on previous research regarding respiratory infections, it was expected that impaired pulmonary functions and reduced exercise capacity would develop due to severe, not mild, influenza or rhinovirus infections [32,33]. Furthermore, it is very important to mention that post-COVID-19 CPETs were performed during the competitive part of the season, when the athletes were expected to be at the peak of their cardiorespiratory fitness, compared to pre-COVID-19 CPETs, which were conducted prior to the start of the season and after a much longer period of rest. Consequently, a significant decrease in VO_2_max could probably be explained by problems at the level of internal respiration. As we mentioned before, the oxygen delivery to working muscles was not pathologically compromised (although it was decreased), which means that the heart and lungs still provided enough oxygen to exert a given intensity. Therefore, the very rapid transition from aerobic to anaerobic metabolism and very low VO_2_max could potentially be explained by the reduced number of mitochondria at the level of the muscle cells and, thus, the inability to adequately utilize oxygen for the creation of ATP. The latest research related to COVID-19 indicates the potential of the disease to damage mitochondria as the main oxygen organelles [34]. It is demonstrated that COVID-19 infection damages the mitochondrion, especially the phosphorylation pathway, which results in abnormal reactive oxygen species production supporting cellular diseases and apoptosis [35]. The abovementioned could explain why the athletes had to consume a significantly higher amount of oxygen for very-low-intensity work (mean VO_2_ achieved at speeds of 8 and 9 km/h) compared to their test results from before contracting COVID-19. Reduced aerobic efficiency and an unfavorable metabolic response for a given intensity were probably the result of mitochondrial damage at the level of muscle cells and impaired internal respiration. Furthermore, the heart rates at VAT and RCP were much lower after COVID-19, which indicates that anaerobic metabolic processes were primarily responsible for obtaining energy during CPET. Therefore, early anaerobic glycolysis (during the early stages of CPET) and high RER max values were the logical consequences of this condition. In other words, the oxygen was there, but it could not be utilized adequately, which led to a whole series of metabolic “disorders” that affected the functional capacity of athletes.

The results of this study confirmed the findings of previous research on the impact of COVID-19 infection on the decline in cardiorespiratory abilities of the young athletic population [16,27,28,31]. However, the main contribution of this study is the evaluation of the reasons that lead to the significantly reduced functional capacity of elite athletes. This study clearly demonstrated that the problem exists at the level of cellular respiration, and that it is necessary to wait for the cell mitosis process with preserved mitochondria to enable the adequate use of oxygen and an increase in cardiorespiratory capabilities.

## 5. Study Limitations and Practical Applications

The study itself has its limitations. The sample size of 27 athletes in this research may be considered small, and it could potentially limit the statistical power and presented results. It is essential to interpret the results with caution and recognize the need for further research with larger sample sizes to establish more precise conclusions. Furthermore, a gender bias should be considered given that the research was conducted on a sample of male athletes. Furthermore, this study was limited to athletes with mild or asymptomatic infection, and further research is needed to assess the impact of severe SARS-CoV-2 infection on cardiorespiratory fitness and possible differences compared to milder forms of COVID-19.

In addition to the limitations mentioned above, the research also has its practical advantages. The main contribution of this study is the evaluation of the reasons that led to the significantly reduced cardiorespiratory fitness of elite athletes. The research showed that the problem exists at the level of cellular respiration and that it is necessary to wait for the cell mitosis process with preserved mitochondria to enable adequate use of oxygen and an increase in cardiorespiratory capabilities. Sports professionals and athletes must take this into account when making return-to-play decisions and pan-training sessions.

## 6. Conclusions

The obtained data showed a significant decrease in oxygen pulse and maximal oxygen consumption, an early transition from aerobic to anaerobic metabolic pathways, increased maximal respiratory exchange ratio, and inefficient use of oxygen at the level of muscle cells. Even though no pathological cardiovascular or respiratory changes were found among athletes after COVID-19, the results showed significantly decreased cardiorespiratory fitness, with an emphasis on internal respiration.

## Figures and Tables

**Table 1:** Pre- and post-COVID-19 CPET measurements related to the cardiorespiratory fitness of professional athletes (n = 27).

Variables	Pre-COVID-19 CPET Test	Post-COVID-19 CPET Test
HR max (bpm)	181.74 ± 7.64	182.67 ± 7.92
HR 1′ (bpm)	157.81 ± 12.32	157.04 ± 11.61
HR 2′ (bpm)	129.33 ± 15.84	128.96 ± 15.12
HR 3′ (bpm)	114.96 ± 13.36	118.48 ± 12.61
VO_2_/HR (mL/beat)	26.27 ± 3.48	23.99 ± 3.21
RFr at VAT (brpm)	29.99 ± 7.72	30.91 ± 7.33
RFr at RCP (brpm)	37.85 ± 7.39	41.81 ± 9.63
BR at VAT (%)	67.30 ± 5.70	62.65 ± 8.16
BR at RCP (%)	51.11 ± 9.62	44.79 ± 9.57
VE/VCO_2_ slope	25.65 ± 3.27	26.29 ± 2.65
OUES	5120.15 ± 847.20	4916.44 ± 788.50
VO_2_max (mL/kg/min)	51.23 ± 6.47	46.2 ± 5.43
Mean VO_2_ at speeds 8 and 9 km/h (mL/kg/min)—steady state	26.24 ± 4.86	28.88 ± 5.14
VO_2_ at VAT (mL/kg/min)	34.33 ± 6.66	29.87 ± 5.88
VO_2_ at VAT (% of VO_2_max)	67.78 ± 9.38	63.95 ± 9.49
HR at VAT (bpm)	146.55 ± 9.12	137.67 ± 20.32
HR at RCP (bpm)	168.93 ± 6.83	161.67 ± 12.56
HR at RER = 1 (bpm)	164.41 ± 10.71	152.30 ± 19.31
RER max	1.14 ± 0.046	1.17 ± 0.051

Data are presented as mean ± SD. Abbreviations: bpm = beats per minute; BR = breathing reserve; brpm = breaths per minute; HR = heart rate; HR 1′, 2′, 3′ = 3 min heart-rate recovery; VO_2_/HR = oxygen pulse; OUES = oxygen uptake efficiency slope; RCP = respiratory compensation point; RER = respiratory exchange ratio; RFr = respiratory frequency; VAT = ventilatory anaerobic threshold; VE/VCO_2_ = ventilatory efficiency; VO_2_ = oxygen uptake; VO_2_max = maximal oxygen uptake.

**Table 2:** Linear regression analysis to predict VO_2_max (mL/kg/min) of VO_2_/HR (mL/beat), VE/VCO_2_, OUES, HR at VAT (bpm), and HR at RCP (bpm) in athletes.

Variables	ẞ Coefficient	Error	*p*-Value
VO_2_/HR	0.160	0.308	0.346
VE/VCO_2_ slope	−0.063	0.297	0.646
OUES	0.117	0.001	0.499
HR at VAT	0.077	0.086	0.723
HR at RCP	0.412	0.124	0.051

R^2^ = 0.337; F = 4.873 (*p* = 0.001). Abbreviations: bpm = beats per minute; HR = heart rate; VO_2_/HR = oxygen pulse; OUES = oxygen uptake efficiency slope; RCP = respiratory compensation point; VAT = ventilatory anaerobic threshold; VE/VCO_2_ = ventilatory efficiency; VO_2_max = maximal oxygen uptake.

## Data Availability

The datasets presented in this article are not readily available because the data are part of an ongoing study. Requests to access the datasets should be directed to corresponding authors.
